# The Efficacy and Safety of Cardiopulmonary Exercise Testing in Advanced Heart Failure Receiving Intravenous Inotropes

**DOI:** 10.3390/jcdd13080354

**Published:** 2026-07-28

**Authors:** Masaki Nakagaito, Teruhiko Imamura, Yuki Hida, Toshihide Izumida, Ryuichi Ushijima, Makiko Nakamura, Koichiro Kinugawa

**Affiliations:** Second Department of Internal Medicine, University of Toyama, Toyama 930-0194, Japan; mgaito128@gmail.com (M.N.);

**Keywords:** advanced heart failure, cardiopulmonary exercise testing, inotrope, heart transplantation

## Abstract

**Background:** Cardiopulmonary exercise testing (CPET) is widely used for prognostic assessment and risk stratification in patients with heart failure (HF). However, its feasibility and clinical utility in patients with advanced HF requiring intravenous inotropic support remain unclear. **Methods:** This observational study included hospitalized patients with HF who received intravenous inotropic therapy and underwent CPET during index hospitalization. The primary outcome was a composite of HF rehospitalization or cardiovascular death. **Results:** A total of 528 patients hospitalized for HF required intravenous inotropic therapy; 50 patients were included. CPET was safely completed in all patients without exercise-related adverse events. The median peak VO_2_ was 12.5 mL/kg/min, and the median VE/VCO_2_ slope was 38.3. During a median follow-up of 762 days, 12 primary events occurred. In univariable analysis, the VE/VCO_2_ slope, O_2_ pulse, and maximal workload were significantly associated with the primary outcome, whereas peak VO_2_ was not. A VE/VCO_2_ slope > 38.2 was associated with a significantly higher 3-year event rate compared with a slope ≤ 38.2 (40% vs. 8%, *p* = 0.021). **Conclusions:** CPET is feasible and safe in highly selected patients with advanced HF requiring inotropic support, and the VE/VCO_2_ slope was associated with clinical outcomes in exploratory univariable analyses.

## 1. Introduction

Currently, cardiopulmonary exercise testing (CPET) is widely utilized not only to assess cardiopulmonary function in patients with heart failure (HF), but also for prognostic evaluation, cardiovascular risk stratification, assessment of therapeutic efficacy, and guidance of cardiac rehabilitation programs [1,2,3,4]. Historically, CPET was primarily used to identify candidates for heart transplantation (HTx) among patients with HF due to left ventricular dysfunction [5]. According to the 2016 International Society for Heart and Lung Transplantation (ISHLT) listing criteria, a peak oxygen uptake (VO_2_) ≤ 12 mL/kg/min is recommended to guide HTx listing in patients receiving β-blocker therapy, whereas a threshold of 14 mL/kg/min may be applied to those intolerant to β-blockers [6].

In recent years, survival among patients with HF with reduced ejection fraction (HFrEF) has improved owing to advances in pharmacological therapies and device-based and interventional therapies [7]. Consequently, patients currently considered for HTx often experience recurrent HF hospitalizations and are frequently unable to perform exercise testing due to severe symptoms at rest or with minimal exertion. Moreover, some candidates require intravenous inotropic support, further limiting the feasibility of CPET. Given that CPET is frequently not feasible in patients with extremely advanced HF, its utility for HTx candidate selection may be reduced in this population.

Conversely, CPET remains a cornerstone in the evaluation of advanced HF and continues to serve as a key complementary tool for risk stratification, including decision-making for HTx or durable ventricular assist device (VAD) in contemporary practice [8,9,10]. Although several studies have reported the utility of CPET in ambulatory patients with advanced HF, there are limited data on CPET performed in hospitalized patients with advanced HF, particularly those requiring intravenous inotropic support [11]. Therefore, the safety and clinical utility of CPET in this population remain unclear.

At our institution, CPET has been performed in patients with advanced HF requiring intravenous inotropic support, including those undergoing continuous inotropic infusion during testing, in accordance with our institutional criteria. In this study, we report our institutional experience with this approach. This investigation may provide clinically relevant insights into the feasibility, safety, and prognostic utility of CPET in this high-risk population, even during ongoing intravenous inotropic support.

## 2. Materials and Methods

### 2.1. Study Design

This study was a single-center, observational study designed to assess the feasibility, safety, and clinical utility of CPET in patients with HF receiving intravenous inotropes. Eligible patients were those hospitalized for worsening chronic HF or acute decompensated HF who were treated with intravenous inotropic agents and underwent at least one CPET during the index hospitalization at a tertiary academic center. Participants received guideline-directed medical therapy for HF, including angiotensin-converting enzyme inhibitors (ACEI), angiotensin receptor blockers (ARB), or angiotensin receptor-neprilysin inhibitors (ARNI), beta-blockers, mineralocorticoid receptor antagonists, sodium-glucose cotransporter 2 inhibitors (SGLT2i), and diuretics, if applicable. The baseline was defined as the date of CPET performance. Baseline characteristics were retrieved on that day, including demographic, laboratory, and medication data. Standard echocardiographic findings were retrieved between admission and CPET.

Patients were excluded from enrollment if they met any of the following criteria: age < 18 years, presence of durable VAD or prior HTx, pregnancy or breastfeeding, severe cognitive impairment or neuromuscular disease, and inability to undergo CPET.

In our institution, CPET was conducted only in participants who fulfilled all of the following criteria: (1) ambulatory within the ward without symptoms, (2) no worsening of subjective symptoms (e.g., dyspnea, fatigue) or physical findings (e.g., edema, pulmonary congestion) of HF, (3) no evidence of worsening HF (e.g., increase in B-type natriuretic peptide levels), (4) absence of uncontrolled arrhythmias, (5) absence of severe valvular disease, and (6) no unstable ischemic heart disease. In addition, CPET was not performed in patients deemed inappropriate by the attending physicians. This study evaluated the safety of CPET under these institutional criteria and examined the association between CPET parameters and subsequent clinical outcomes.

The study was conducted in accordance with the Declaration of Helsinki, and prior informed consent was diligently obtained from all individuals participating in this investigative endeavor. The local Institutional Ethics Board of Toyama University Hospital approved the study protocol (#R2015154).

### 2.2. Exercise Testing

CPET was conducted using a system consisting of a leg ergometer (Konami Sports Life, Tokyo, Japan), a breath-by-breath gas analyzer (Minato Medical Science, Osaka, Japan), and 12-lead electrocardiography for continuous monitoring (Fukuda Denshi, Tokyo, Japan). Our exercise protocol was based on the recommendations of the American Heart Association [12]. CPET began with 3 min of seated rest on the cycle ergometer, followed by a symptom-limited exercise test. Each test employed a ramp protocol with incremental workload, set at 7, 10, and 15 W/min to yield a fatigue-limited exercise duration of 8 to 12 min. All tests were supervised by a cardiologist.

During CPET, the following parameters were directly measured: oxygen uptake (VO_2_), carbon dioxide production (VCO_2_), minute ventilation (VE), and respiratory rate. Blood pressure and heart rate were measured at one-minute intervals during the test. Derived indices included the respiratory exchange ratio (RER), ventilatory efficiency (VE/VCO_2_ slope), anaerobic threshold (AT), and peak O_2_ pulse.

The VE/VCO_2_ slope was calculated from breath-by-breath data of VE and VCO_2_ plotted throughout exercise using linear regression analysis. AT was identified by a nonlinear increase in the VCO_2_–VO_2_ slope (V-slope method). Exercise testing was terminated when patients reached volitional exhaustion or at the discretion of the supervising physician based on safety concerns. A Borg scale questionnaire was administered after the test to assess symptom perception.

### 2.3. Outcomes

The primary outcome was a composite of unplanned hospitalization for HF or death from cardiovascular causes. Implantation of a VAD or HTx was considered equivalent to cardiovascular death. The secondary endpoint was all-cause mortality, including cardiovascular death, non-cardiovascular death, HTx, or VAD implantation.

Patients were censored at the time of last follow-up if they did not experience the primary outcome. Data for patients who did not have the endpoint event were censored on the last day they were known to have been free of the event. All patients were followed until the end of the observation period or for 36 months.

### 2.4. Statistical Analyses

All statistical computations were executed employing JMP^®^ 18 (SAS Institute Inc., Cary, NC, USA). Significance was ascribed to outcomes featuring a two-sided *p*-value of <0.050. Continuous variables were conveyed as medians in conjunction with interquartile ranges (IQRs), and the appropriate statistical assessments were conducted employing the Wilcoxon test. Categorical data were articulated in terms of counts and corresponding percentages, and the appropriate statistical assessments were conducted employing the chi-square test.

The Kaplan–Meier method was used to estimate the cumulative 3-year incidence of clinical events, and differences were assessed using the log-rank test. Univariable Cox regression analysis was used to calculate the hazard ratio to assess the influence of various parameters on clinical outcomes. For univariable analysis, we selected values of patients’ characteristics, etiology of HF, major markers of HF, and CPET parameters. Receiver operating characteristic (ROC) analysis was carried out to determine the cut-off value of the parameters used in the Cox regression analysis.

## 3. Results

### 3.1. Follow-Up and Patient Characteristics

From January 2020 to October 2025, a total of 528 patients hospitalized for HF required intravenous inotropic therapy. Among these, 53 patients underwent CPET during the index hospitalization. One patient underwent CPET but was excluded from this study because frailty precluded application of any workload using the ramp protocol. Two patients were excluded because they had previously undergone durable VAD implantation.

**Figure 1:**
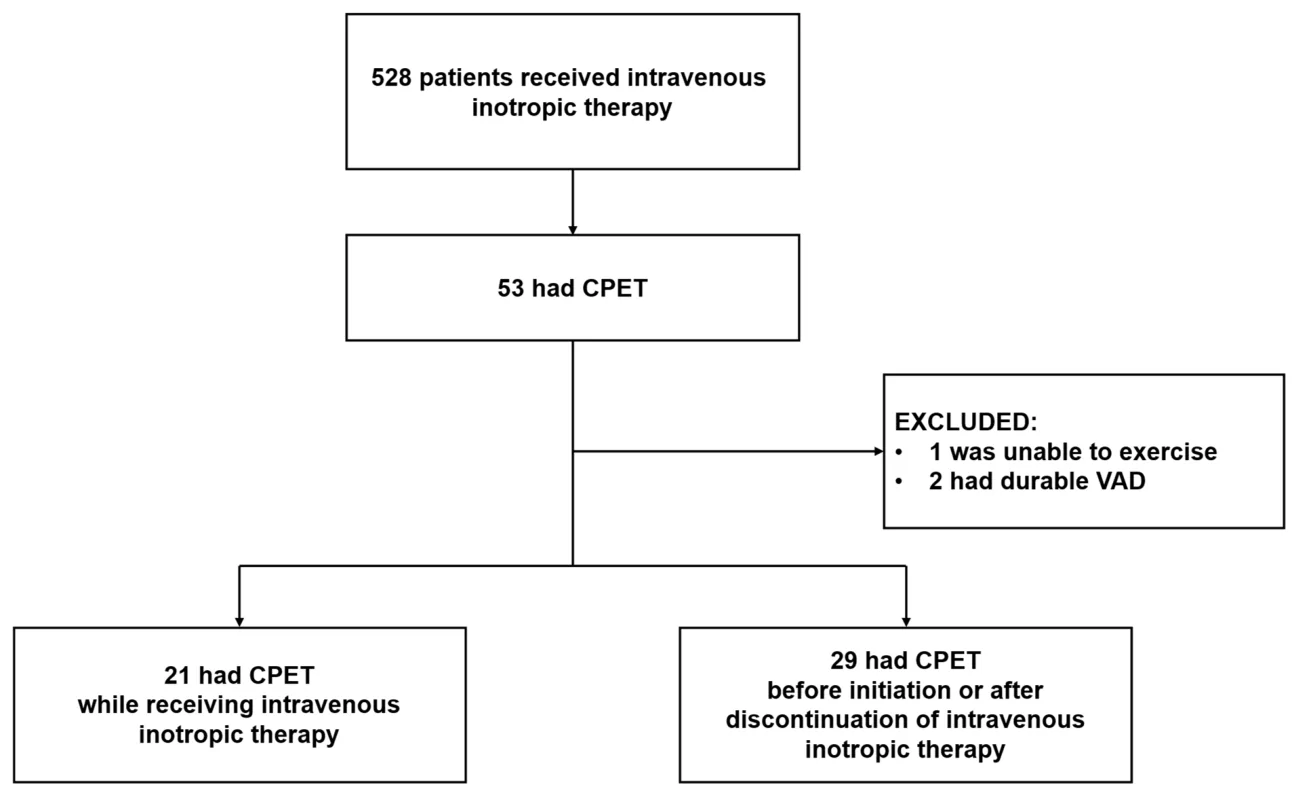
Patient diagram. CPET, cardiopulmonary exercise test; VAD, ventricular assist device.

A total of 50 patients were included in this study. Twenty-one patients who underwent CPET while receiving intravenous inotropic therapy were defined as the inotrope group, whereas 29 patients, comprising three who underwent CPET before inotropic therapy and 26 who underwent CPET after discontinuation of inotropic therapy, were defined as the non-inotrope group (Figure 1).

Table 1 lists the baseline characteristics. The median age was 58 (49–67) years and 72% were male. The median left ventricular ejection fraction was 32 (22–43) %. Ischemic etiology was present in 14 patients (28%), and dilated cardiomyopathy in 17 patients (34%). Diabetes mellitus was present in 21 patients (42%). Medical therapy was optimized, with 100% of patients taking ACEI, ARB, or ARNI and 98% taking beta-blockers. Baseline plasma B-type natriuretic peptide level was 188 (120–339) pg/mL.

All patients in the inotrope group received dobutamine, and milrinone was co-administered in two cases. The median dose of dobutamine was 2.0 μg/kg/min. Patients in the inotrope group had lower systolic blood pressure and higher hemoglobin levels compared with those in the non-inotrope group. In addition, the prevalence of dilated cardiomyopathy was higher in the inotrope group.

All patients in the inotrope group were weaned off intravenous inotropes at a median of 30 (14–48) days after the CPET. Although 3 patients underwent implantation of a durable VAD during the index hospitalization, all patients were discharged home ambulatory following a median of 47 (24–68) days of hospitalization. The length of hospital stay was significantly longer in the inotrope group than in the non-inotrope group (64 (35–92) days versus 37 (21–53) days, *p* = 0.010). Following index discharge, patients were followed for a median period of 762 (346–1095) days. While the median follow-up period did not differ significantly between the two groups, follow-up duration varied among individuals.

### 3.2. Cardiopulmonary Exercise Test Parameters

CPET was completed safely without any exercise-related worsening of HF, ischemic symptoms, or new-onset arrhythmias requiring additional treatment. Results of the CPET variables are shown in Table 2, stratified by inotrope and non-inotrope groups. Overall, the median peak VO_2_ was 12.5 (10.6–15.4) mL/kg/min, and the median VE/VCO_2_ slope was 38.3 (34.1–46.8). Most CPET parameters were not significantly different between the inotrope and non-inotrope groups. The only parameter that differed significantly was the peak RER, which was higher in the inotrope group than in the non-inotrope group.

### 3.3. Clinical Outcomes

There were 12 primary composite events throughout the 3-year follow-up (9 HF hospitalizations and 3 durable VAD implantations). All three patients who underwent implantation of a durable VAD belonged to the inotrope group. Table 3 presents the results of the analysis of factors associated with the primary outcome. In univariable Cox proportional hazards analysis, the VE/VCO_2_ slope, peak O_2_ pulse, and maximal workload were significantly associated with HF hospitalization or cardiovascular death, whereas peak VO_2_ was not associated with the primary outcome. The use of intravenous inotropic agents at the time of CPET was not significantly associated with the primary outcome.

**Figure 2:**
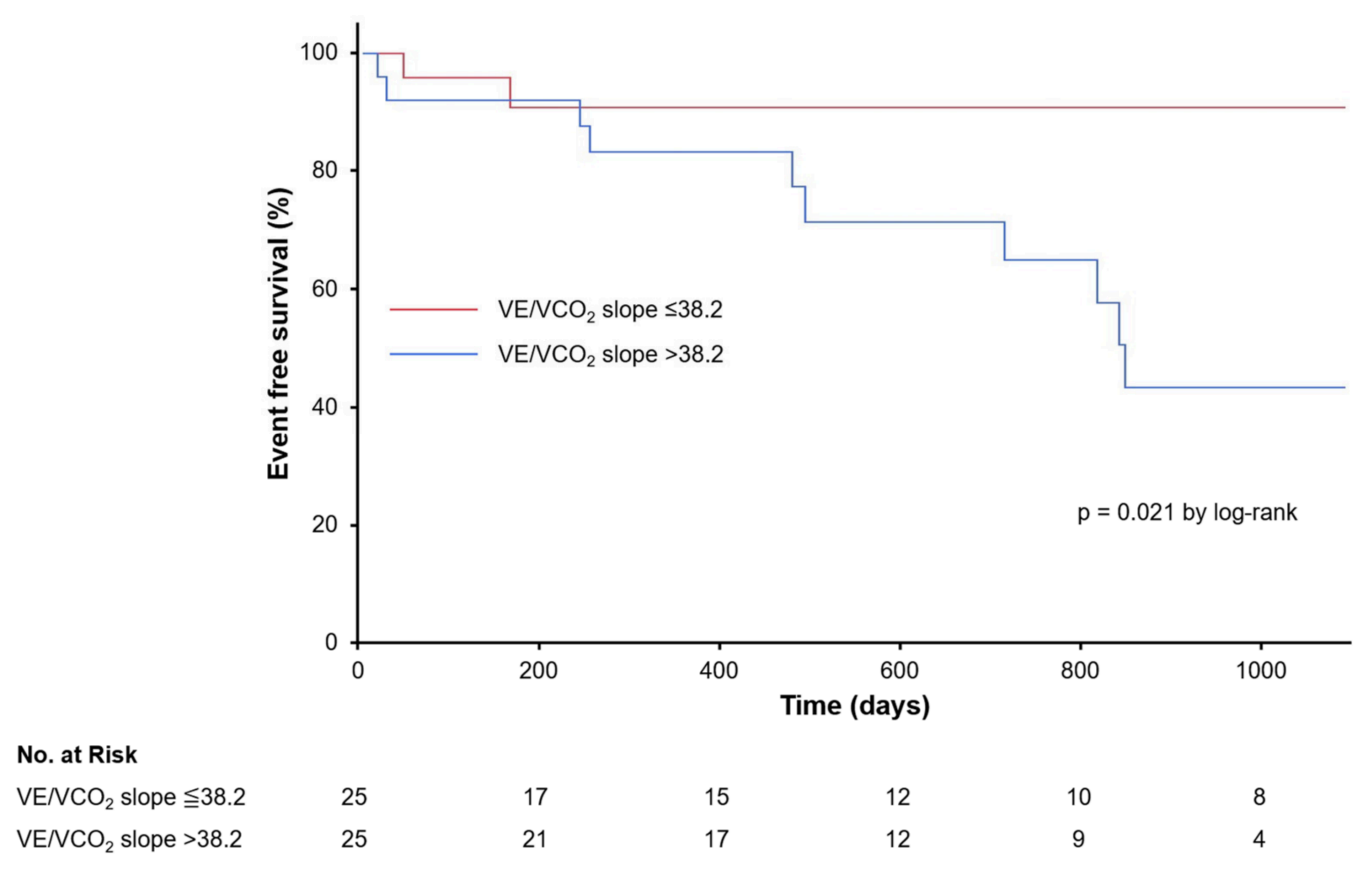
Kaplan–Meier curves for hospitalization for heart failure or cardiovascular death with VE/VCO_2_ slope threshold of 38.2.

A cut-off of baseline VE/VCO_2_ slope to predict the primary endpoint was 38.2 (area under the curve 0.738, sensitivity 0.605, and specificity 0.833). The results from Kaplan–Meier analysis revealed a 3-year primary endpoint of 40% in patients with VE/VCO_2_ > 38.2 and 8% in those with VE/VCO_2_ ≤ 38.2 (*p* = 0.021 by log-rank; Figure 2).

Seven patients experienced secondary outcome events, comprising three cardiovascular deaths, two non-cardiovascular deaths, and three VAD implantations. Univariable Cox proportional hazards analysis showed that age and maximal workload achieved during CPET were associated with the secondary outcome (Table 4).

## 4. Discussion

In this study, we evaluated the feasibility, safety, and clinical utility of CPET in patients with advanced HF receiving intravenous inotropic therapy. CPET was performed only after hemodynamic stabilization using a cycle ergometer with an individualized ramp protocol. Under our institutional criteria, CPET was safely completed in all eligible patients, with no adverse events, and all patients were ultimately discharged.

Notably, CPET parameters were largely comparable between patients undergoing testing during inotropic therapy and those assessed before initiation or after discontinuation of inotropes, suggesting that ongoing inotropic support did not substantially influence CPET-derived parameters. Furthermore, CPET parameters, particularly indices of ventilatory efficiency, were significantly associated with the composite endpoint of HF hospitalization or cardiovascular death in this high-risk population, whereas peak VO_2_ was not associated with outcomes.

### 4.1. Feasibility of CPET

The present study demonstrates that CPET can be safely performed in carefully selected patients with advanced HF receiving intravenous inotropic support, provided that strict selection criteria and expert supervision are applied. To our knowledge, this is the first study to specifically evaluate CPET in this high-risk population. Safety is the foremost concern in this population, as these patients are often hemodynamically unstable and presumed to be at high risk during exercise testing. Nonetheless, in our cohort, no adverse events occurred, highlighting that CPET is feasible even under continuous inotropic therapy when conducted within a structured protocol and under the close supervision of experienced cardiologists. Furthermore, the peak RER observed in the present study was clearly higher than that reported in previous studies evaluating CPET in patients with advanced HF [11]. This finding suggests that the enrollment criteria in our study appropriately selected patients who were able to tolerate maximal exercise testing. Therefore, the CPET data obtained in this study are likely to be reliable for the assessment of exercise capacity and prognostic evaluation. On the other hand, among the 528 patients hospitalized for HF who required intravenous inotropic therapy, CPET was performed in only 53 patients. Therefore, our findings should be interpreted in the context of this highly selected study population. The present results support the safety and feasibility of CPET only in carefully selected and clinically stabilized patients with advanced HF and should not be generalized to all patients requiring inotropic support.

In the present study, one patient was excluded from CPET because severe lower-limb muscle weakness prevented the application of any exercise workload. One of the limitations of the present study is the difficulty in accurately assessing baseline exercise capacity prior to CPET, particularly in patients receiving intravenous inotropic support, in whom conventional functional assessment may be unreliable. Such patients may be identifiable prior to testing by assessing physical performance, such as with the Short Physical Performance Battery (SPPB) or objective measurements of lower-limb muscle strength [13]. Incorporating these assessments into our eligibility criteria may allow more appropriate patient selection and further enhance the safety of CPET in patients with advanced HF.

Previous studies of intravenous inotropic therapy have primarily enrolled patients with worsening HF, a population in whom assessment of functional capacity is often challenging. However, in real-world clinical practice, we frequently encounter situations in which it is necessary to assess the functional capacity of patients who are receiving long-term intravenous inotropic therapy or who are dependent on inotropes. Indeed, in this setting, prognostic assessment and the decision regarding HTx listing are limited to the clinical outcome, which is influenced by several uncontrolled variables, and to a few measured parameters and mainly hemodynamic variables and BNP values. Our findings suggest that CPET may provide complementary physiological and prognostic information in carefully selected patients receiving continuous intravenous inotropic therapy. However, the safety findings of the present study should be considered preliminary and interpreted within the context of our institution’s eligibility criteria and management protocol. The absence of observed adverse events should not be interpreted as definitive evidence of the safety or broader applicability of CPET in this high-risk population. Larger prospective multicenter studies are warranted to establish the safety and clinical utility of CPET in patients receiving intravenous inotropic support.

### 4.2. CPET Parameters with or Without Inotropic Therapy

CPET parameters were generally similar regardless of inotropic therapy status. Previous studies have shown that intravenous inotropic agents can acutely improve cardiac output and peripheral perfusion in patients with advanced HF, which may lead to transient improvements in exercise capacity [14,15]. Indeed, a previous study demonstrated that in patients hospitalized with HF, treatment with the inodilator levosimendan increased peak VO_2_ and reduced the VE/VCO_2_ slope [16]. Therefore, CPET parameters obtained during inotropic support may theoretically overestimate intrinsic exercise capacity.

Although most CPET parameters did not differ according to the use of inotropic therapy, peak RER was significantly higher in the inotrope group. Intravenous inotropic therapy may influence the RER through changes in cardiac output, metabolic rate, and exercise capacity, potentially modifying the ventilatory and metabolic responses during CPET. Importantly, because comparisons were made between different patients, the direct physiological effects of inotropic therapy could not be fully isolated. A within-subject design, with CPET performed both during and off inotropic support, would be required to more precisely define these effects.

The non-inotrope group included both patients who underwent CPET before the initiation of inotropic therapy and those who underwent CPET after discontinuation of inotropic support. We acknowledge that these patients may have been in different clinical conditions at the time of CPET. However, our intention was not to compare these clinically distinct populations. Rather, we aimed to provide a descriptive comparison of CPET parameters according to the presence or absence of inotropic support at the time of testing. On this basis, these patients were combined into a single non-inotrope group for the present analysis.

### 4.3. Clinical Impact of CPET

This study demonstrated that CPET parameters were more strongly associated with clinical outcomes than etiology or major markers of HF. These findings are consistent with the recommendations of the ISHLT guidelines, which emphasize the importance of CPET parameters, particularly peak VO_2_ and the VE/VCO_2_ slope, for risk stratification and for determining candidacy for advanced therapies such as HTx or durable VAD implantation. In the present study, the VE/VCO_2_ slope was significantly associated with adverse clinical outcomes, whereas peak VO_2_ was not. This finding may be explained by several factors specific to patients with advanced HF requiring intravenous inotropic therapy. Peak VO_2_ is highly dependent on patient effort and peripheral factors such as skeletal muscle function, frailty, and exercise motivation, all of which are frequently impaired in patients with advanced HF. Furthermore, intravenous inotropic agents may transiently augment cardiac output and peripheral perfusion, potentially improve exercise capacity and attenuate the relationship between peak VO_2_ and long-term prognosis. Consequently, peak VO_2_ may not accurately reflect the severity of underlying circulatory dysfunction in this population.

Because CPET findings themselves may have influenced decisions regarding HTx listing or durable VAD implantation, we performed an additional sensitivity analysis excluding durable VAD implantation and HTx from the primary composite endpoint. The analysis demonstrated that the VE/VCO_2_ slope remained significantly associated with the revised endpoint consisting of HF hospitalization and cardiovascular death only (Table S1). However, given the small number of outcome events, this sensitivity analysis may have been underpowered, and the results should therefore be interpreted with caution.

In contrast, the VE/VCO_2_ slope reflects ventilatory inefficiency associated with impaired pulmonary perfusion, ventilation-perfusion mismatch, and abnormal chemoreflex sensitivity, which are key pathophysiological features of advanced HF [17]. Because this parameter is less dependent on maximal effort and more closely reflects underlying cardiopulmonary pathophysiology, it may provide more robust prognostic information in patients receiving inotropic support. In clinical settings where peak VO_2_ may be influenced by inotropic therapy or reduced exercise tolerance, ventilatory efficiency parameters such as VE/VCO_2_ slope may therefore provide complementary and potentially more reliable prognostic information for identifying patients who require timely consideration of advanced therapeutic strategies. Indeed, several studies have demonstrated that a VE/VCO_2_ slope of >35 is of greater prognostic value than a peak VO_2_ of <14 mL/kg/min [18,19,20,21]. In the present study, the ROC-derived cut-off value of the VE/VCO_2_ slope for predicting the primary outcome was 38.2, which is slightly higher than the threshold of approximately 35 commonly used for risk stratification in advanced HF and recommended by the ISHLT guidelines [6]. However, the cut-off value identified in this study represents an exploratory finding derived from a small cohort. Accordingly, the optimal threshold should be validated in larger, independent cohorts before being considered for clinical application.

From a clinical perspective, CPET performed under inotropic support may assist in tracking functional status, evaluating therapeutic response (including inotrope weaning), and guiding decisions regarding HTx or VAD candidacy. However, larger studies focusing specifically on patients undergoing CPET during inotropic therapy are needed to validate these findings and refine prognostic thresholds.

### 4.4. Limitations

This study has several limitations. First, it was a single-center observational study with a relatively small sample size, particularly in the subgroup receiving inotropic support. Because of the limited number of primary outcome events, it was not feasible to construct a robust multivariable model. CPET findings may differ depending on the underlying etiology of HF and disease duration [22]. Given the small cohort in the present study, the potential influence of these factors could not be fully accounted for and may have affected our findings. Second, the oxygen uptake efficiency slope (OUES), an established prognostic marker in advanced HF, could not be assessed due to technical limitations. Third, inotropic regimens were heterogeneous, reflecting real-world practice. Because CPET was performed under varying agents and infusion rates, the specific effects of individual drugs could not be distinguished from underlying disease severity. Finally, CPET findings may have influenced clinical decision-making, including listing for HTx or initiation of VAD therapy, introducing potential incorporation bias in outcome assessment.

## 5. Conclusions

In conclusion, CPET performed during continuous intravenous inotropic therapy is feasible in carefully selected patients with advanced HF. The results of this study suggest that an elevated VE/VCO_2_ slope is associated with adverse cardiac events in patients with advanced HF. Although CPET parameters should be interpreted with caution in this setting, submaximal indices and measures of ventilatory efficiency may provide complementary prognostic information to support clinical assessment.

## Figures and Tables

**Table 1:** Baseline characteristics.

	Total (*n* = 50)	Inotrope (*n* = 21)	Non-Inotrope (*n* = 29)	*p*-Value
Age, years	58 (49–67)	58 (49–66)	57 (49–72)	0.753
Male, n (%)	36 (72)	16 (76)	20 (69)	0.574
Body mass index, kg/m^2^	20.5 (19.2–23.8)	21.5 (19.1–25.7)	20.4 (19.2–22.5)	0.467
Systolic blood pressure, mmHg	95 (83–107)	88 (81–98)	105 (84–113)	0.006 *
Heart rate, beats per minute	75 (68–84)	75 (63–80)	71 (64–83)	0.187
Diabetes mellitus, n (%)	21 (42)	11 (52)	10 (35)	0.206
Ischemic etiology, n (%)	14 (28)	4 (19)	10 (35)	0.230
Dilated cardiomyopathy, n (%)	17 (34)	13 (62)	4 (14)	<0.001 *
Atrial fibrillation, n (%)	12 (24)	5 (24)	7 (24)	0.979
Left ventricular ejection fraction, %	32 (22–43)	31 (21–40)	32 (26–47)	0.279
Hemoglobin, g/dL	12.7 (10.8–14.3)	13.2 (11.7–15.1)	11.7 (10.4–13.3)	0.043 *
Serum albumin, g/dL	3.5 (3.1–3.8)	3.5 (3.1–4.0)	3.5 (3.2–3.8)	0.882
Serum sodium, mEq/L	139 (137–140)	139 (138–141)	139 (137–140)	0.197
Serum potassium, mEq/L	4.3 (4.0–4.8)	4.3 (4.1–4.7)	4.2 (4.0–4.9)	0.867
eGFR, mL/min/1.73 m^2^	54.8 (44.1–68.6)	48.6 (44.7–65.4)	57.0 (41.3–73.6)	0.569
Uric acid, mg/dL	4.5 (3.7–6.3)	3.9 (3.5–5.4)	4.6 (3.8–6.5)	0.109
Total bilirubin, mg/dL	0.6 (0.4–0.8)	0.6 (0.4–0.8)	0.6 (0.4–0.8)	0.819
Plasma BNP, pg/mL	188 (120–339)	229 (134–364)	167 (91–320)	0.238
Treatment				
Dobutamine, μg/kg/min	0 (0–2.0)	2.0 (1.8–3.0)	0 (0–0)	<0.001 *
Beta-blockers, n (%)	49 (98)	21 (100)	28 (97)	0.390
ACEI/ARB/ARNI, n (%)	50 (100)	21 (100)	29 (100)	NA
MRA, n (%)	46 (92)	20 (95)	26 (90)	0.473
SGLT2 inhibitors, n (%)	38 (76)	18 (86)	20 (69)	0.171
Loop diuretics, n (%)	24 (48)	11 (52)	13 (45)	0.598
Dose of loop diuretics, mg/day	0 (0–10)	10 (0–20)	0 (0–10)	0.472

eGFR, estimated glomerular filtration rate; BNP, B-type natriuretic peptide; ACEI, angiotensin converting enzyme inhibitors; ARB, angiotensin receptor blockers; ARNI, angiotensin receptor-neprilysin inhibitors; MRA, mineralocorticoid receptor antagonists; SGLT2 inhibitors, sodium-glucose cotransporter 2 inhibitors. * *p* < 0.050.

**Table 2:** Baseline cardiopulmonary exercise variables.

	Total (*n* = 50)	Inotrope (*n* = 21)	Non-Inotrope (*n* = 29)	*p*-Value
Peak VO_2_, mL/kg/min	12.5 (10.6–15.4)	13.1 (11.1–15.7)	12.2 (10.4–15.5)	0.549
VE/VCO_2_ slope	38.3 (34.1–46.8)	43.0 (35.2–47.9)	38.1 (33.2–44.3)	0.157
AT, mL/kg/min	8.5 (7.5–9.4)	8.4 (7.7–9.2)	8.5 (7.2–9.4)	0.714
Peak RER	1.19 (1.11–1.25)	1.23 (1.15–1.31)	1.14 (1.09–1.23)	0.018 *
Peak O_2_ pulse, mL	6.5 (5.7–8.5)	6.7 (5.4–8.9)	6.5 (5.9–8.2)	0.829
HRR	13 (5–21)	13 (8–24)	13 (3–20)	0.271
Peak systolic blood pressure, mmHg	117 (102–128)	114 (102–123)	117 (107–131)	0.267
Peak heart rate, beats per minute	116 (102–132)	120 (103–133)	110 (97–132)	0.461
Maximal workload, W	61 (45–82)	68 (46–88)	57 (43–80)	0.275
Borg score at peak exercise	14 (13–17)	14 (13–17)	14 (13–16)	0.520

VO_2_, oxygen uptake; VE, minute ventilation; VCO_2_, volume of exhaled carbon dioxide; AT, anaerobic threshold; RER, respiratory exchange ratio; HRR, heart rate recovery. * *p* < 0.050.

**Table 3:** Variables associated with heart failure hospitalization or cardiovascular death.

Variables	Outcome (−) (*n* = 38)	Outcome (+) (*n* = 12)	Hazard Ratio (95% CI)	*p*-Value
Inotrope group, n	16 (42)	5 (42)	1.21 (0.38, 3.84)	0.742
Age, years	56 (49–66)	65 (44–70)	1.02 (0.98, 1.06)	0.424
Body mass index, kg/m^2^	20.6 (19.4–26.1)	19.9 (17.5–23.1)	0.87 (0.72, 1.01)	0.078
Ischemic etiology, n	12 (32)	2 (17)	0.43 (0.09, 1.95)	0.271
LVEF, %	32 (22–45)	34 (21–42)	1.01 (0.97, 1.04)	0.671
Hemoglobin, g/dL	12.7 (10.7–14.3)	12.3 (11.4–14.3)	1.01 (0.80, 1.25)	0.912
eGFR, mL/min/1.73 m^2^	57.0 (44.7–71.6)	48.5 (33.9–67.8)	0.99 (0.97, 1.02)	0.551
ln BNP	5.15 (4.56–5.74)	5.62 (5.01–6.12)	1.42 (0.66, 2.89)	0.367
Peak VO_2_, mL/kg/min	13.4 (11.1–15.9)	11.3 (9.7–12.4)	0.83 (0.65, 1.01)	0.066
VE/VCO_2_ slope	37.5 (33.3–46.0)	45.5 (38.7–48.8)	1.09 (1.01, 1.20)	0.027 *
AT, mL/kg/min	8.6 (7.8–9.4)	7.7 (6.5–8.8)	0.68 (0.36, 1.22)	0.197
Peak RER	1.19 (1.11–1.25)	1.20 (1.09–1.27)	2.28 (0.02, 166.69)	0.718
Peak O_2_ pulse, mL	6.8 (5.7–8.8)	6.0 (4.2–6.5)	0.60 (0.39, 0.88)	0.007 *
HRR	14 (7–23)	9 (3–17)	0.96 (0.89, 1.03)	0.271
Peak SBP, mmHg	117 (102–132)	115 (102–121)	0.97 (0.94, 1.00)	0.115
Peak HR, beats per minute	119 (103–132)	109 (92–123)	0.98 (0.95, 1.01)	0.178
Maximal workload, W	65 (48–86)	46 (38–65)	0.97 (0.93, 1.00)	0.022 *
Borg score at peak exercise	14 (13–17)	14 (12–15)	0.90 (0.66, 1.20)	0.492

LVEF, left ventricular ejection fraction; eGFR, estimated glomerular filtration rate; BNP, B-type natriuretic peptide; VO_2_, oxygen uptake; VE, minute ventilation; VCO_2_, volume of exhaled carbon dioxide; AT, anaerobic threshold; RER, respiratory exchange ratio; HRR, heart rate recovery; SBP, systolic blood pressure; HR, heart rate. * *p* < 0.050.

**Table 4:** Variables associated with all-cause mortality.

Variables	Outcome (−) (*n* = 43)	Outcome (+) (*n* = 7)	Hazard Ratio (95% CI)	*p*-Value
Inotrope group, n	18 (42)	3 (43)	1.29 (0.29, 5.82)	0.737
Age, years	56 (48–66)	70 (65–75)	1.10 (1.03, 1.19)	0.004 *
Body mass index, kg/m^2^	21.0 (19.3–25.8)	20.4 (18.8–20.6)	0.84 (0.62, 1.03)	0.104
Ischemic etiology, n	11 (26)	3 (43)	1.68 (0.38, 7.52)	0.498
LVEF, %	32 (21–42)	35 (27–47)	1.02 (0.98, 1.06)	0.311
Hemoglobin, g/dL	12.7 (10.8–14.7)	11.5 (10.4–13.0)	0.77 (0.51, 1.07)	0.133
eGFR, mL/minute/1.73 m^2^	56.9 (45.1–68.2)	36.3 (33.1–73.2)	0.99 (0.96, 1.02)	0.492
ln BNP	5.28 (4.69–5.75)	5.09 (4.96–6.18)	1.08 (0.40, 2.90)	0.875
Peak VO_2_, mL/kg/min	13.3 (10.5–15.8)	11.8 (11.3–12.4)	0.87 (0.64, 1.11)	0.282
VE/VCO_2_ slope	38.2 (33.8–46.6)	42.6 (35.3–49.1)	1.03 (0.94, 1.15)	0.545
AT, mL/kg/min	8.5 (7.7–9.4)	7.1 (6.4–8.9)	0.74 (0.44, 1.21)	0.238
Peak RER	1.19 (1.10–1.25)	1.20 (1.11–1.27)	2.68 (0.01, 982.59)	0.748
Peak O_2_ pulse, mL/beat	6.7 (5.6–8.7)	5.9 (5.7–6.4)	0.70 (0.42, 1.08)	0.110
HRR	13 (6–21)	13 (3–25)	0.99 (0.91, 1.06)	0.851
Peak SBP, mmHg	116 (102–130)	117 (79–121)	0.97 (0.93, 1.01)	0.183
Peak HR, beats per minute	117 (102–132)	103 (82–128)	0.97 (0.93, 1.01)	0.205
Maximal workload, W	66 (46–85)	45 (38–48)	0.95 (0.90, 0.99)	0.022 *
Borg score at peak exercise	15 (13–17)	14 (13–14)	1.00 (0.69, 1.40)	0.993

LVEF, left ventricular ejection fraction; eGFR, estimated glomerular filtration rate; BNP, B-type natriuretic peptide; VO_2_, oxygen uptake; VE, minute ventilation; VCO_2_, volume of exhaled carbon dioxide; AT, anaerobic threshold; RER, respiratory exchange ratio; HRR, heart rate recovery; SBP, systolic blood pressure; HR, heart rate. * *p* < 0.050.

## Data Availability

The data presented in this study are available on request from the corresponding author. The data are not publicly available due to privacy.

## Supplementary Materials

The following supporting information can be downloaded at: https://www.mdpi.com/article/10.3390/jcdd13080354/s1, Table S1: Variables associated with heart failure hospitalization or cardiovascular death (excluding durable VAD implantation or HTx).
