# Supplementary material for: The Efficacy and Safety of Cardiopulmonary Exercise Testing in Advanced Heart Failure Receiving Intravenous Inotropes
Source: J Cardiovasc Dev Dis. 2026 Jul 28;13(8):354. doi: 10.3390/jcdd13080354 (PMC13513396; doi:10.3390/jcdd13080354)
Supplement: Supplementary file 1 [file jcdd-13-00354-s001.zip › jcdd-4426591-supplementary.pdf]

Table S1. Variables associated with heart failure hospitalization or cardiovascular death (excluding durable VAD implantation or HTx)

Variables	Outcome (−) (*n* = 41)	Outcome (+) (*n* = 9)	Hazard Ratio (95% CI)	p value
Inotrope group, n	19 (46)	2 (22)	0.51 (0.11, 2.48)	0.407
Age, years	57 (50-67)	64 (40-72)	1.01 (0.97, 1.05)	0.750
Body mass index, kg/m^2	20.6 (19.4-24.9)	19.4 (17.9-23.0)	0.88 (0.69, 1.03)	0.124
Ischemic etiology, n	13 (32)	1 (11)	0.25 (0.03, 2.02)	0.194
LVEF, %	32 (22-43)	33 (20-48)	1.01 (0.97, 1.05)	0.547
Hemoglobin, g/dL	12.6 (10.7-14.0)	12.8 (11.6-15.2)	1.06 (0.82, 1.34)	0.633
eGFR, mL/minute/1.73m^2	56.9 (44.7-71.5)	47.6 (34.7-67.4)	0.99 (0.96, 1.02)	0.620
ln BNP	5.12 (4.63-5.77)	5.71 (5.19-6.13)	1.47 (0.59, 3.28)	0.392
Peak VO_2, mL/kg/min	13.1 (11.2-15.8)	9.8 (9.4-13.1)	0.80 (0.59, 1.02)	0.069
VE/VCO_2 slope	37.5 (33.4-46.1)	46.6 (39.1-49.6)	1.10 (1.00, 1.23)	0.040*
AT, mL/kg/min	8.5 (7.7-9.4)	7.7 (6.7-9.1)	0.99 (0.98, 1.00)	0.012*
Peak RER	1.19 (1.11-1.25)	1.15 (1.02-1.26)	0.27 (0.01, 47.9)	0.632
Peak O_2 pulse, mL/beat	6.7 (5.7-8.7)	6.4 (4.1-6.6)	0.62 (0.36, 0.95)	0.027*
HRR	13 (7-24)	4 (3-16)	0.95 (0.85, 1.02)	0.183
Peak SBP, mmHg	117 (102-131)	113 (103-120)	0.96 (0.90, 1.00)	0.073
Peak HR, beats per minutes	120 (103-133)	107 (92-119)	0.97 (0.93, 1.00)	0.086
Maximal workload, W	61 (47-86)	42 (34-68)	0.96 (0.92, 1.00)	0.037*
Borg score at peak exercise	13 (12-15)	14 (13-17)	0.80 (0.53, 1.15)	0.236

VAD, ventricular assist device; HTx, heart transplantation; LVEF, left ventricular ejection fraction; eGFR, estimated glomerular filtration rate; BNP, B-type natriuretic peptide; VO_2, oxygen uptake; VE, minute ventilation; VCO_2, volume of exhaled carbon dioxide; AT, anaerobic threshold; RER, respiratory exchange ratio; HRR, heart rate recovery; SBP, systolic blood pressure; HR, heart rate.

*p <0.050.
